# Surgical Interval as a Risk Factor for Postoperative Complications After Repeated Lung Cancer Resection: A Retrospective Cohort Study

**DOI:** 10.1016/j.atssr.2026.03.004

**Published:** 2026-03-21

**Authors:** Yuka Kadomatsu, Keita Nakanishi, Harushi Ueno, Taketo Kato, Shota Nakamura, Tetsuya Mizuno, Toyofumi Fengshi Chen-Yoshikawa

**Affiliations:** 1 Department of Thoracic Surgery, Nagoya University Graduate School of Medicine, Nagoya, Japan

## Abstract

**Background:**

With improved survival after initial surgery, repeated pulmonary resections are increasingly performed. However, the clinical effect of the interval between operations remains unclear.

**Methods:**

This study retrospectively analyzed data from patients who underwent only contralateral repeated pulmonary resection for lung cancer at a single institution between 2002 and 2024, assessing 30-day postoperative complications. Surgical interval was categorized as ≤3 months and >3 months. Multivariable logistic regression was employed to identify risk factors for morbidity.

**Results:**

The final cohort comprised 246 contralateral second resections in 123 patients. The mean surgical interval was 40.2 months (median, 27 months). Approximately 40% of second resections occurred within 12 months of the first, half of which were within 3 months. Postoperative complications developed in 25 (20.3%; n = 25/123) patients who were frequently male, had a lower body mass index, and more commonly underwent anatomic resection. Multivariable analysis revealed age (odds ratio [OR], 1.15; 95% CI, 1.04-1.28), male sex (OR, 0.09; 95% CI, 0.02-0.40), anatomic resection (OR, 11.0; 95% CI, 2.08-58.3), and ≤3-month surgical interval (OR, 3.36; 95% CI, 1.01-11.3) as independently associated with postoperative complications. The overall morbidity rate was significantly higher in patients with a ≤3-month interval compared with longer intervals (36.0% [n = 9/25] vs 16.3% [n = 16/98]; *P* = .029).

**Conclusions:**

Contralateral repeated pulmonary resection is feasible, but a ≤3-month surgical interval is associated with greater postoperative morbidity. The surgical interval should be considered an important factor in operative planning.

**In Short**

▪ Repeated pulmonary resection for second primary lung cancer is generally safe, although the optimal interval between surgeries remains unclear.
▪ A surgical interval of ≤3 months was independently associated with an increased risk of postoperative complications.

The incidence of second primary lung cancer has been increasingly recognized in recent years owing to early detection with advanced imaging modalities, improvements in surgical safety that enable repeated pulmonary resection, and standardized postoperative surveillance leading to earlier identification of additional lesions.^1^ Patient-reported outcome studies have revealed that recovery of certain functions and quality of life domains may take up to 6 months after an initial lung resection.^2^ However, the effect of the interval between the first and second surgeries on postoperative outcomes remains unclear as the surgical interval has not been a major focus of previous investigations.^3^^,^^4^

This study evaluated the association of the duration between successive pulmonary resections with postoperative complications after the second surgery in patients undergoing repeated lung cancer surgery. Study findings are expected to provide evidence to guide optimal management strategies for second pulmonary resections.

## Patients and Methods

### Ethical Statement

This retrospective study enrolled all patients who underwent curative-intent surgery for lung cancer at Nagoya University from January 1, 2002, to June 30, 2024, at a single hospital. The institutional review board of Nagoya University School of Medicine approved the study protocol (2020-0375, October 14, 2020), and the requirement for informed consent was waived because of the retrospective study design.

### Patient Selection and Data Collection

We included patients who underwent initial R0 resection for lung cancer at our institution during the study period who met the following criteria: the initial surgery was an anatomic resection; and patients underwent 2 or more surgeries for lung cancer. We restricted our analysis to the second and subsequent surgeries for patients who had undergone 3 or more resections.

We excluded patients with lung tumors who had undergone wedge resection only or incomplete resection. Furthermore, patients who underwent ipsilateral repeated pulmonary resections were excluded because preliminary analysis revealed that a small number of ipsilateral cases were required to enable robust statistical evaluation. Moreover, previous studies have demonstrated higher complication rates after second ipsilateral resections compared with contralateral resections.^5^

### Data Collection

Clinical data were obtained from a prospectively maintained institutional database and verified by attending physicians. Variables including demographic characteristics, comorbidities, Charlson comorbidity index, preoperative pulmonary function, surgical procedures, operative time, blood loss, length of hospital stay, and postoperative outcomes were collected.

### Variable Definition

Postoperative complications were evaluated following the criteria defined by the National Clinical Database of Japan.^6^ In the National Clinical Database system, postoperative complications are recorded on the basis of predefined clinical criteria and the requirement for therapeutic intervention, rather than being graded according to severity classification systems. Major complications were defined as an air leak that lasted >7 days or required treatment, including chemical pleurodesis and repeated drainage, and pneumonia with severe inflammation confirmed by radiologic imaging. The surgical interval was defined as the period between the first and second surgeries. The primary outcome was any complication occurring within 30 days after the second surgery.

### Statistical Methods

Descriptive results were expressed as counts and percentages for categorical variables and as means and SD for continuous variables by Pearson *χ*^2^ or Fisher exact test and the Student *t*-test, respectively.

Multivariable logistic regression analysis was conducted to estimate adjusted odds ratios and 95% CIs. Covariates included in the model were age, sex, impaired pulmonary function, Charlson comorbidity index, surgical procedure (anatomic resection or not), and tumor size, which were selected a priori on the basis of their clinical relevance and previous literature reporting associations with postoperative complication incidences.^7^

Statistical Package for the Social Sciences version 30.0 software (IBM) was used for statistical analyses. All *P* values were 2 sided, and *P* values of <.05 indicated statistical significance.

## Results

**Figure 1:**
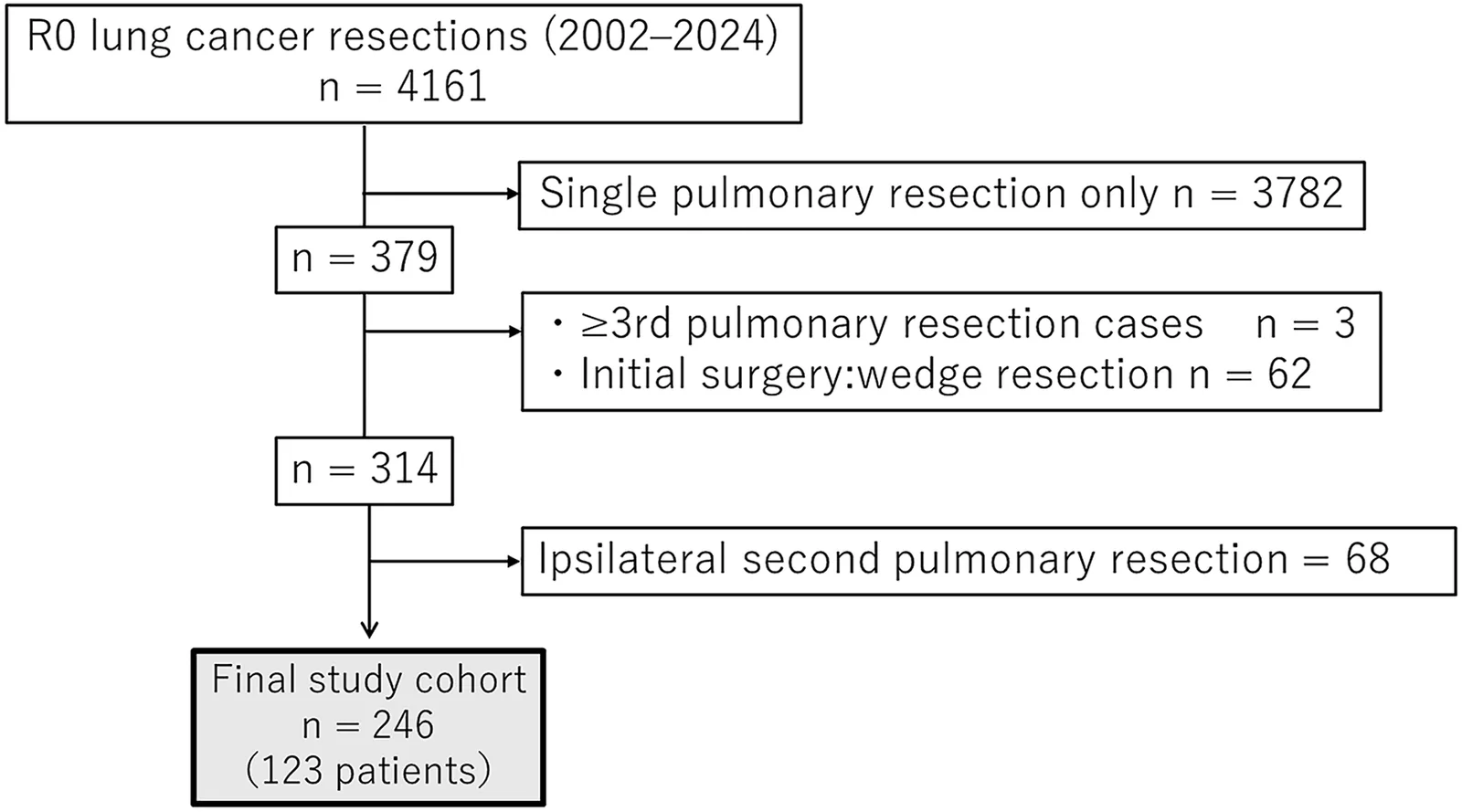
Flow diagram of case selection.

The final analysis included 246 contralateral second pulmonary resections in 123 patients (Figure 1).

**Figure 2:**
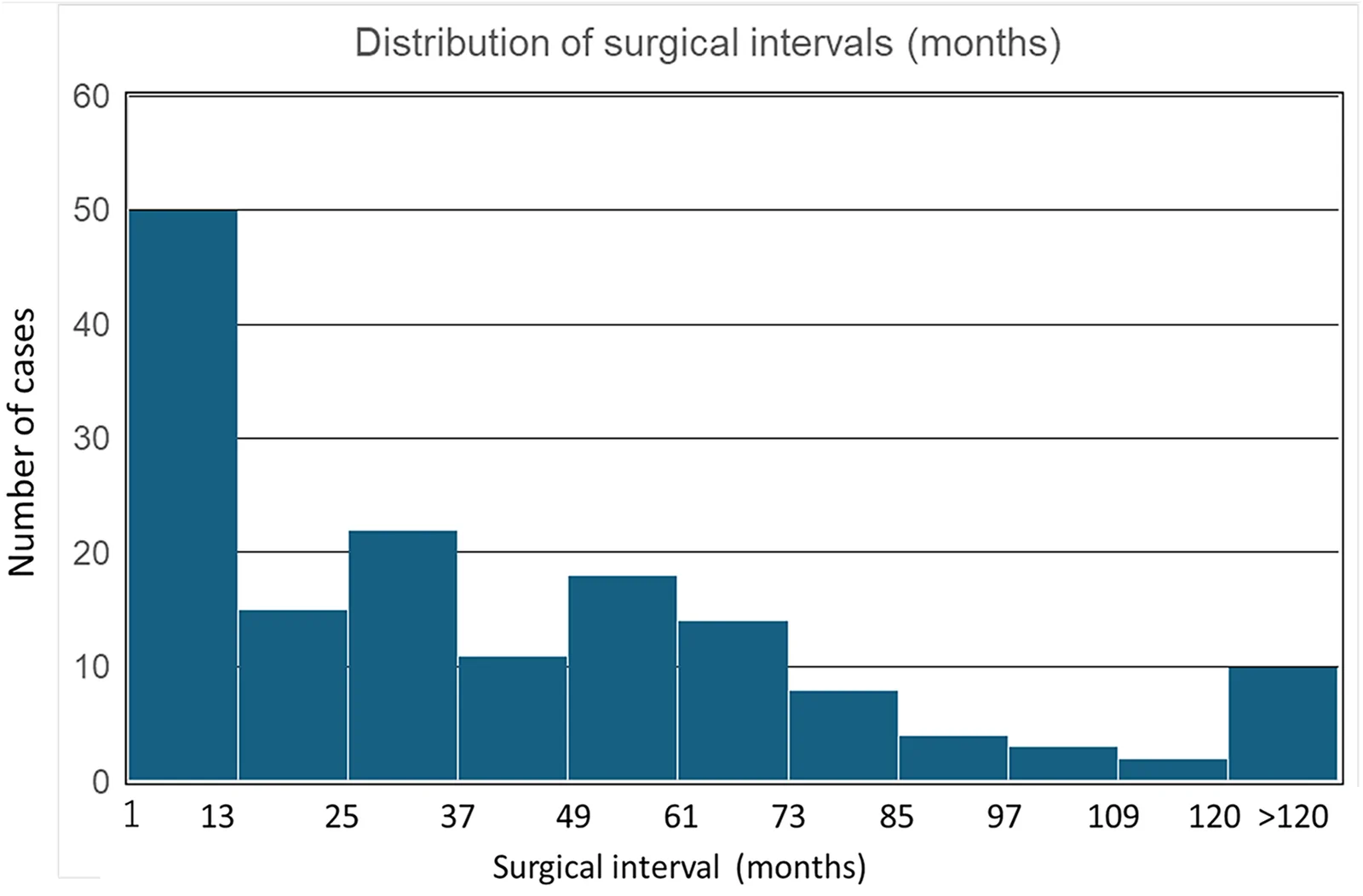
Distribution of surgical intervals between the first and second pulmonary resections.

Postoperative complications were observed in 25 (20.3%) cases. Table 1 summarizes the baseline characteristics stratified by the presence of complications. Compared with patients without complications, those with complications were more frequently male, had a lower body mass index, and more commonly underwent anatomic resection. Other baseline characteristics, including age, impaired pulmonary function, comorbidities, tumor size, and operative time, did not significantly differ between the groups. Baseline characteristics stratified by surgical interval (≤3 months vs >3 months) are additionally presented in Supplemental Table 1. Figure 2 illustrates the distribution of surgical intervals between the first and second pulmonary resections. The mean interval between the 2 surgeries was 40.2 months (median, 27 months). Approximately 40% of patients underwent a second pulmonary resection within 1 year of the previous procedure, half of which were performed within 3 months.

**Table 1:** Baseline Characteristics of Patients With Contralateral Repeated Pulmonary Resection Stratified by Postoperative Complications

Variable	Complication + (n = 25)	Complication − (n = 98)	*P* Value
Age, y	72.7 ± 6.0	70.1 ± 7.8	.082
Male	20 (80.0)	46 (46.9)	.003^a^
BMI, kg/m^2^	21.8 ± 4.5	22.4 ± 2.8	.004^a^
Impaired pulmonary function	17 (68.0)	55 (56)	.306
Comorbidities			
CCI ≥3	13 (52.0)	32 (33)	.079
History of malignant disease	8 (32.0)	23 (23)	.381
Diabetes mellitus	7 (28.0)	17 (17.3)	.261
Arrhythmia	1 (4.0)	3 (3.1)	1
Surgical procedure			.034^a^
Wedge resection	3 (12.0)	33 (33.7)	
Segmentectomy or lobectomy	22 (88.0)	65 (66.3)	
Tumor size, mm	23.5 ± 8.7	19.9 ± 9.7	.399
Surgical approach VATS or RATS	11 (44.0)	66 (68.8)	.022^a^
Operative time, min	155.6 ± 40.5	128.3 ± 57.9	.074
Length of hospital stay, d	20.7 ± 15.1	9.1 ± 3.5	<.001^a^

Categorical variables are presented as number of patients (%). Continuous variables are presented as mean ± SD.

Pulmonary dysfunction was defined as vital capacity <80% or forced expiratory volume in 1 second/forced vital capacity <70%.

BMI, body mass index; CCI, Charlson comorbidity index; RATS, robot-assisted thoracic surgery; VATS, video-assisted thoracic surgery.

a Independently associated with postoperative complications.

On multivariable analysis, a shorter surgical interval of ≤3 months was independently associated with a higher risk of postoperative complications (odds ratio, 3.36; 95% CI, 1.01-11.3; *P* = .049). Older male patients who underwent anatomic resection were more likely to experience postoperative complications (Table 2).

**Table 2:** Multivariable Logistic Regression Analysis of Risk Factors for Postoperative Complications

Variable	Category	OR	95% CI	*P* Value
Age	Per 1-year increase	1.15	1.04-1.28	.006^a^
Sex	Female (reference: male)	0.09	0.02-0.40	.001^a^
Impaired pulmonary function	Yes (reference: no)	0.56	0.16-1.98	.363
CCI	>3 (reference: ≤3)	0.89	0.48-1.63	.696
Surgical procedure	Anatomic resection (reference: wedge resection)	11	2.08-58.32	.005^a^
Tumor size	Per 1-mm increase	1.02	0.96-1.07	.556
Interval ≤3 months	Yes (reference: >3 months)	3.36	1.01-11.3	.049^a^

CCI, Charlson comorbidity index; OR, odds ratio.

a Independently associated with postoperative complications.

The incidence of postoperative complications stratified on the basis of surgical interval is shown in Supplemental Table 2. The overall complication rate was significantly higher in patients with an interval of ≤3 months compared with those with longer intervals (36.0% [n = 9/25] vs 16.3% [n = 16/98]; *P* = .029). Higher numerical rates of prolonged air leak and inflammatory pulmonary complications, including pneumonia, were observed in the early interval group despite no statistically significant difference in the specific complication between the groups.

## Comment

This study aimed to investigate the effect of surgical interval on postoperative complications in patients undergoing a second surgery for lung cancer. We retrospectively analyzed data from patients who underwent repeated lung cancer surgeries, assessing clinical characteristics, surgical procedures, and postoperative complications. Repeated surgeries accounted for 7.5% of all lung cancer surgeries during the study period. Of these, we focused on contralateral surgeries and assessed the association between surgical interval and postoperative complications. The evaluation of surgical intervals in 1-year increments revealed that approximately half of the second surgeries were performed within 1 year of the first operation, and of these, nearly half were conducted within 3 months. Multivariable analysis revealed that a surgical interval of ≤3 months was independently associated with an increased risk of postoperative complications in addition to older age, male sex, and anatomic resection.

Several reports have also indicated that ipsilateral re-resection, particularly completion pneumonectomy, carries a substantially increased risk of postoperative complications and requires careful perioperative management.^4^ Therefore, this study excluded patients who underwent ipsilateral second resections and focused exclusively on contralateral repeated pulmonary resections.

In this study, the surgical interval of 3 months was selected as a clinically meaningful threshold. Evidence from patient-reported outcome studies indicates that the timing of full recovery after lung cancer surgery differs across reports but most consistently shows that patients have not achieved full recovery within the first 3 months.^2^ The addition of the burden of a second surgical intervention under such conditions may increase vulnerability to complications. Previous investigations have revealed that postoperative cytokine release, including interleukin 6, is associated with adverse outcomes after pulmonary resection.^8^ Balancing the potential for disease progression with the risks associated with operating before adequate physiologic recovery, we adopted ≤3 months as the cutoff for the surgical interval in this study. Our results indicate that a surgical interval of ≤3 months is associated with a higher complication risk. Therefore, surgical interval should be recognized as an important factor in perioperative risk assessment, and the timing of repeated resection should be determined on the basis of overall clinical judgment.

This study has some limitations. First, the analysis was based on our institutional database; thus, patients who had previously undergone lung resection at other hospitals may not have been recorded. Second, the study period spanned nearly 2 decades, during which perioperative management and surgical techniques have evolved. In addition, unmeasured factors, including technical complexity and other procedure-related characteristics that are difficult to quantify, may have influenced both the choice of surgical procedure and the risk of postoperative complications. Third, the tumor stage could not be analyzed as the TNM criteria underwent substantial revisions during the study period. Instead, tumor size was included as a surrogate variable. Fourth, we focused exclusively on contralateral resections to minimize heterogeneity; however, the results may not be generalizable to ipsilateral repeated resections, which are known to carry a higher perioperative risk. Finally, the retrospective, single-center design inherently introduces the risk of selection bias, whereas the modest sample size, particularly in the ≤3-month interval group, resulted in wide CIs and limited the precision of effect estimates. Accordingly, the observed relationships in this study should be interpreted as associations rather than as evidence of a causal relationship.

In conclusion, a surgical interval of ≤3 months between repeated lung cancer surgeries was associated with an increased risk of postoperative complications. Surgical interval should be considered an important factor in perioperative risk assessment.

## Data Availability

The data underlying this article cannot be shared publicly to maintain the privacy of individuals who participated in the study. Data supporting the findings of this study are available from the corresponding author upon reasonable request.

## References

[1] Motono N., Ishikawa M., Iwai S. (2021). Individualization of risk factors for postoperative complication after lung cancer surgery: a retrospective study. BMC Surg.

[2] Kadomatsu Y., Oga T., Ota A. (2025). Analysis of the changes in health-related quality of life and employment status after surgery in patients with lung cancer: a single-center longitudinal study. Gen Thorac Cardiovasc Surg.

[3] Abid W., Seguin-Givelet A., Brian E. (2021). Second pulmonary resection for a second primary lung cancer: analysis of morbidity and survival. Eur J Cardiothorac Surg.

[4] Hamaji M., Ali S.O., Burt B.M. (2015). A meta-analysis of resected metachronous second non-small cell lung cancer. Ann Thorac Surg.

[5] Komatsu H., Izumi N., Tsukioka T. (2023). Restrictive ventilatory impairment as a poor prognostic factor in patients who undergo surgical resection for metachronous second primary lung cancer. Ann Thorac Cardiovasc Surg.

[6] Endo S., Ikeda N., Kondo T. (2017). Model of lung cancer surgery risk derived from a Japanese nationwide web-based database of 78 594 patients during 2014-2015. Eur J Cardiothorac Surg.

[7] Ferguson M.K., Gaissert H.A., Grab J.D., Sheng S. (2009). Pulmonary complications after lung resection in the absence of chronic obstructive pulmonary disease: the predictive role of diffusing capacity. J Thorac Cardiovasc Surg.

[8] Ramer-Quinn D.S., Baker R.A., Sanders V.M. (1997). Activated T helper 1 and T helper 2 cells differentially express the beta_2_-adrenergic receptor: a mechanism for selective modulation of T helper 1 cell cytokine production. J Immunol.

